# Supplementary figures and images for: Metabolic and Immune Markers for Precise Monitoring of COVID-19 Severity and Treatment
Source: Front Immunol. 2022 Jan 12;12:809937. doi: 10.3389/fimmu.2021.809937 (PMC8790058; doi:10.3389/fimmu.2021.809937)

# Supplementary Figure 1

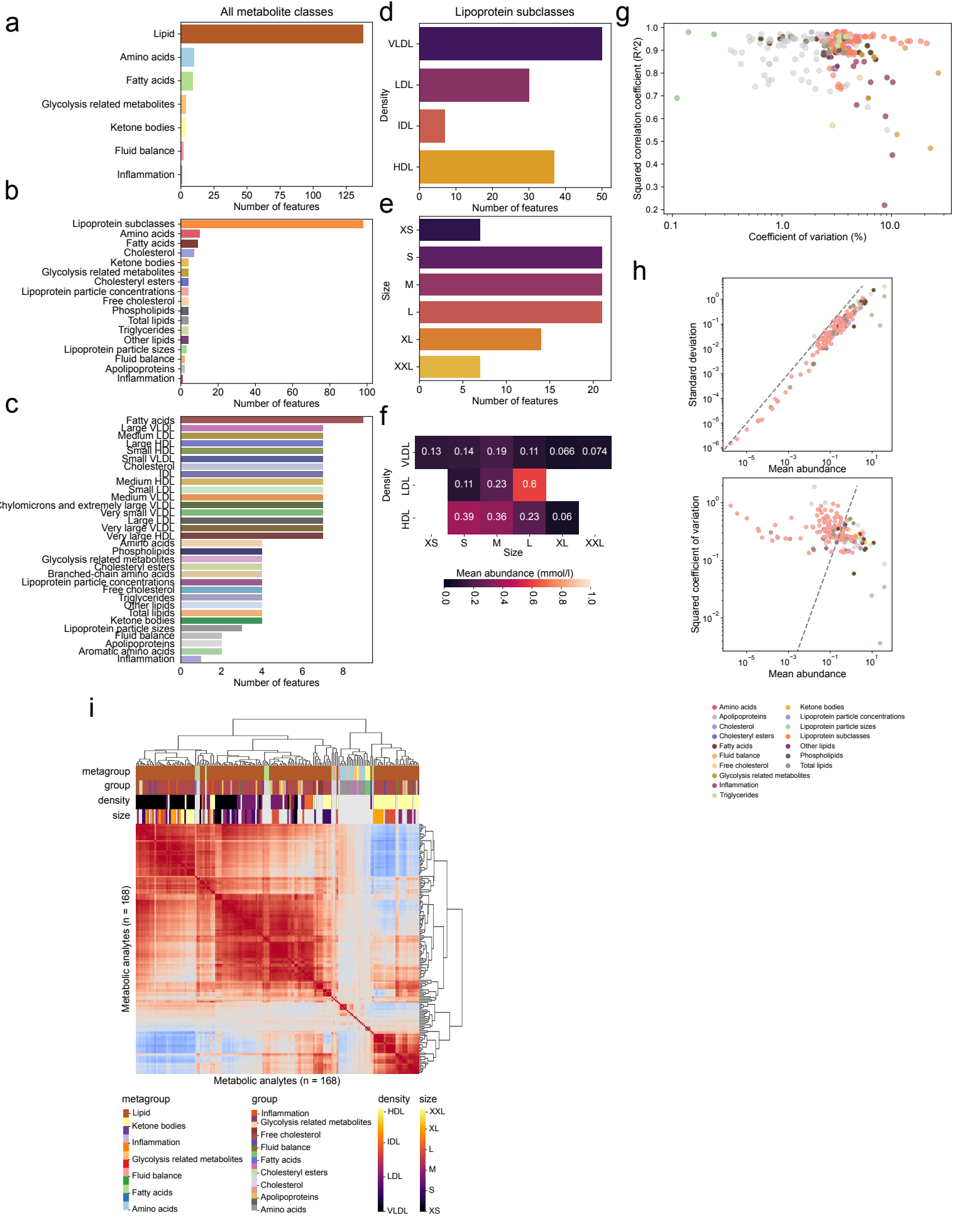

Supplement: Supplementary Figure 1 — Characterization of the NMR metabolomics panel. (A–C) Composition of the panel dependent on the biophysical characteristics of the analytes from A to C with increased granularity. (D, E) Composition of the lipoprotein particle variables in the panel depending on their density (D) and size (E). (F) Absolute abundance of metabolites depending on their density or size. (G) Measures of reproducibility and signal-to-noise for all metabolites in the panel. (H) Relationship between mean and variance for all variables in the panel. (I) Pair-wise correlation of metabolite abundance. [file Image_1.pdf]

# Supplementary Figure 2

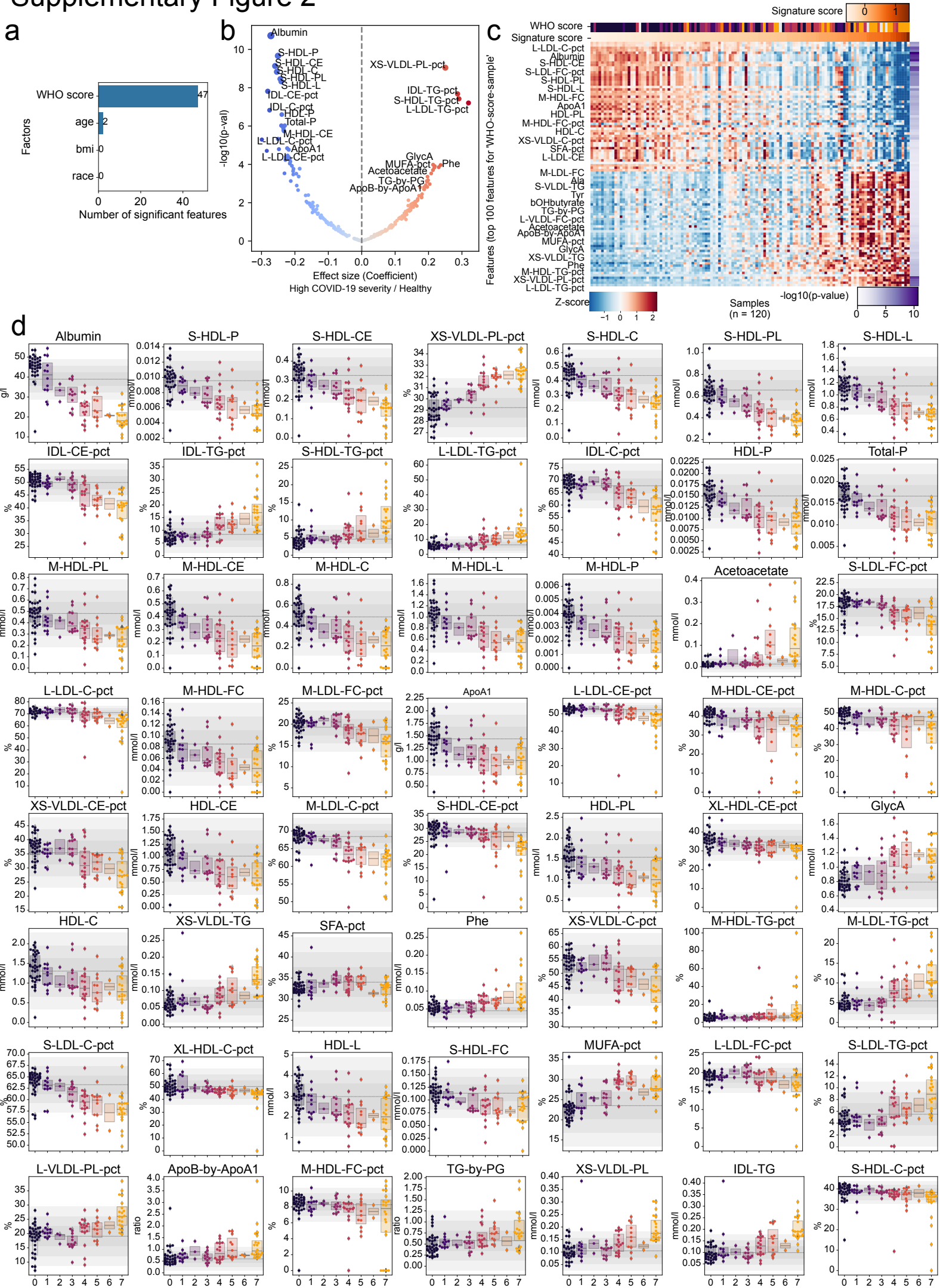

Supplement: Supplementary Figure 2 — Metabolic changes associated with COVID-19 severity. (A) Number of significant (p < 0.05 FDR) variables for a joint model of COVID-19 severity, patient age, BMI, and race. (B) Volcano plot of changes in metabolites associated with COVID-19 severity. (C) Heatmap of relative metabolite abundance for all samples where the axes have been sorted by the amount of change. (D) Abundance of metabolites with significant association with COVID-19 severity depending on WHO score. [file Image_2.pdf]

# Supplementary Figure 3

a

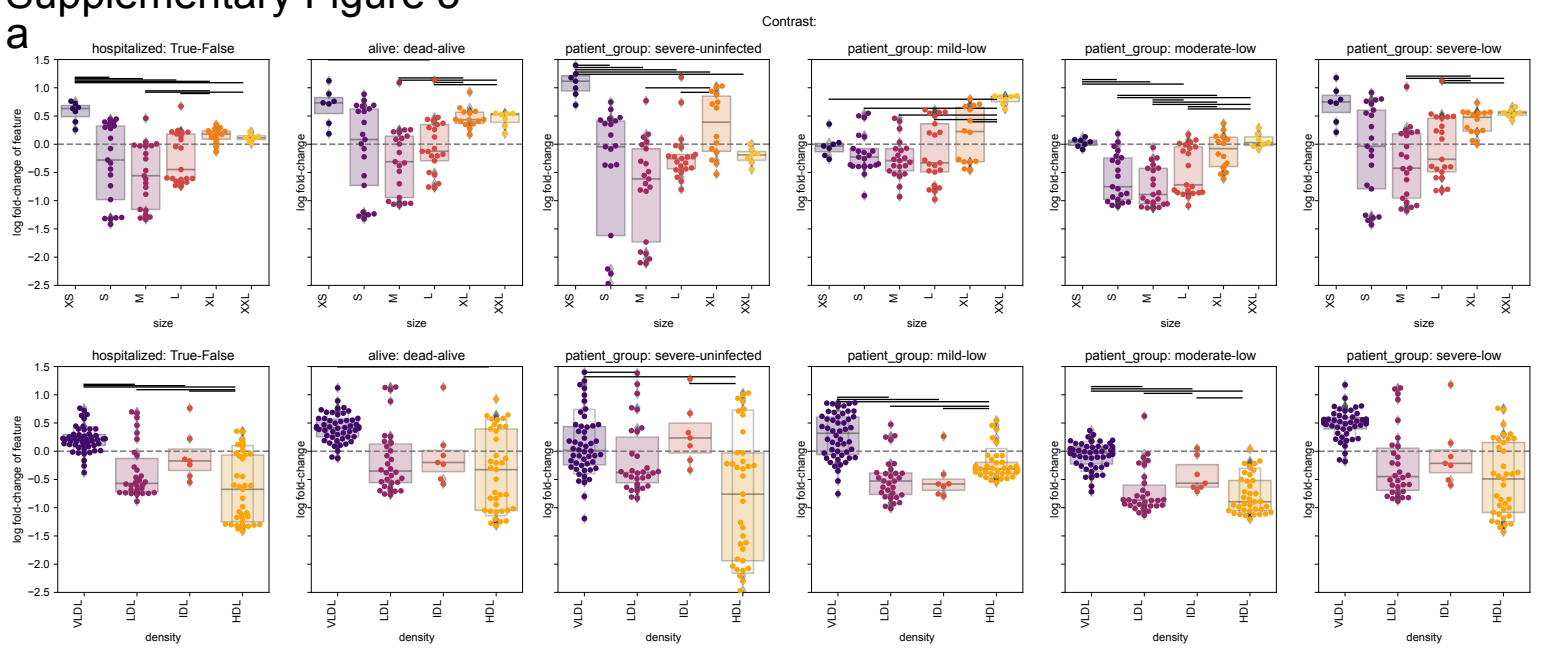

b

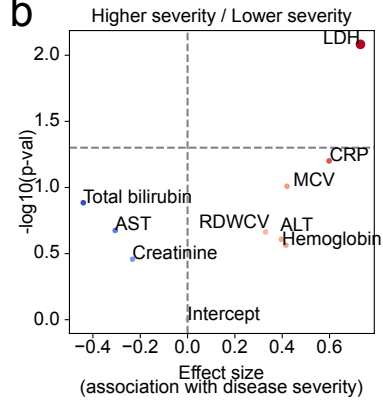

c

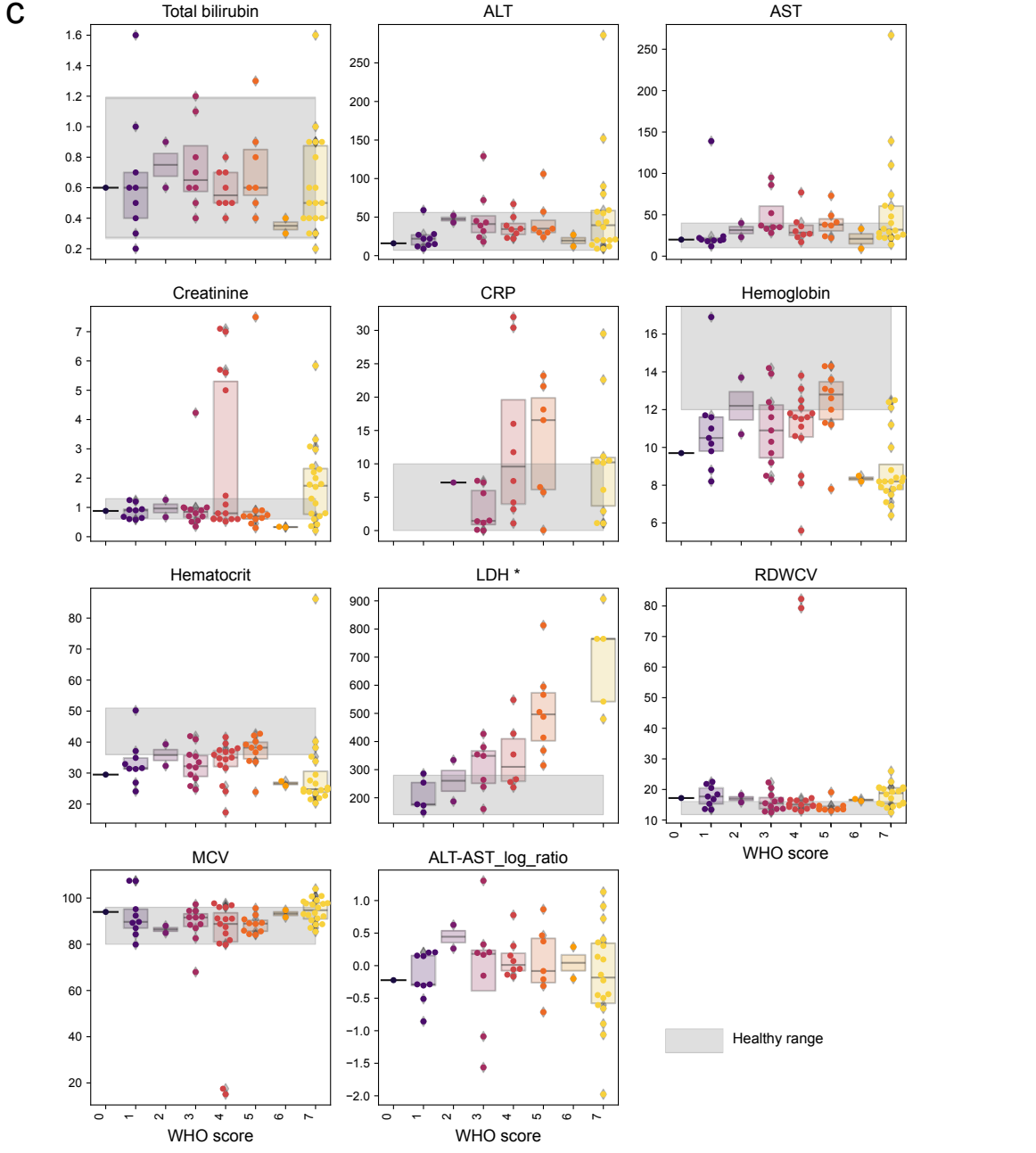

Supplement: Supplementary Figure 3 — Metabolic and clinical association of disease severity. (A) Distribution of log fold-changes in lipoprotein particle metabolites depending on their size (upper row) or density (lower row). The coefficients represent the change associated with hospitalization, death and disease severity. (B) Volcano plot of clinical variables associated with COVID-19 severity in our cohort. (C) Distribution of clinical parameters in the samples dependent of COVID-19 severity. The horizontal grey areas represent a healthy range for each parameter. [file Image_3.pdf]

Supplementary Figure 4

a

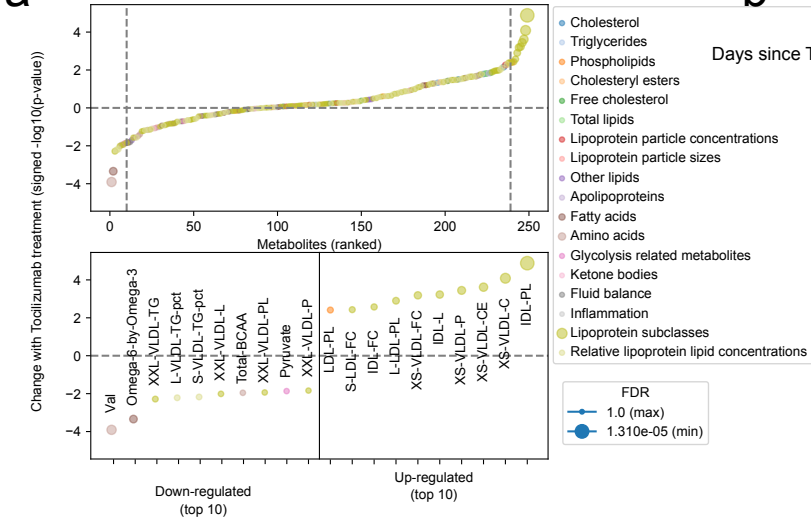

b

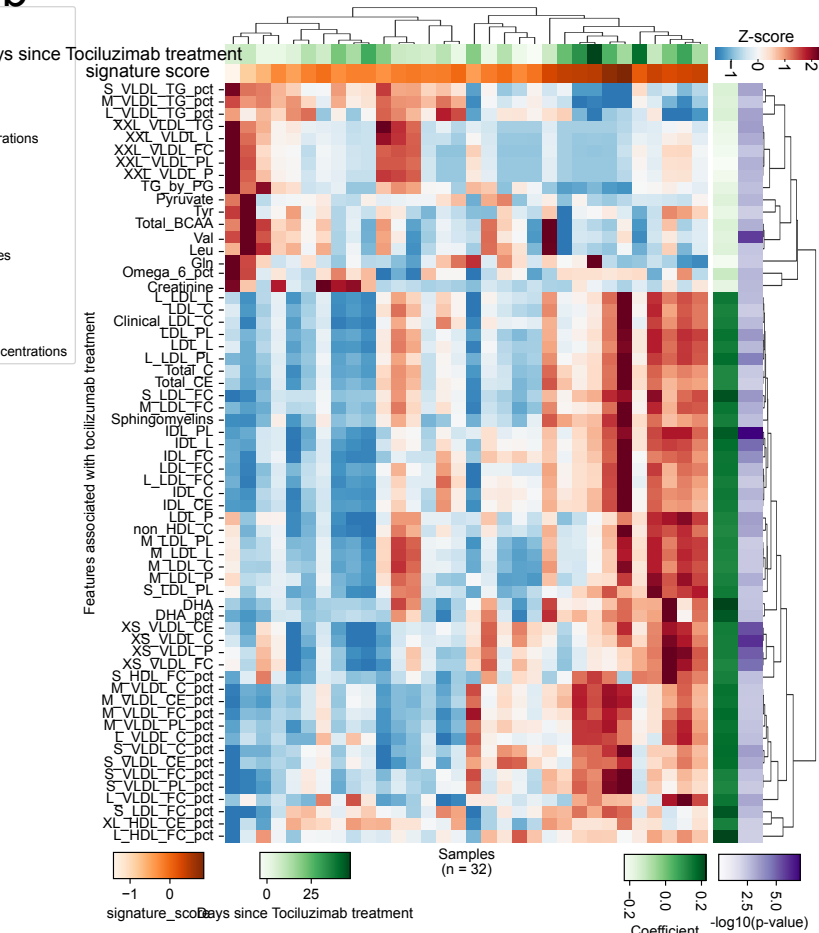

c

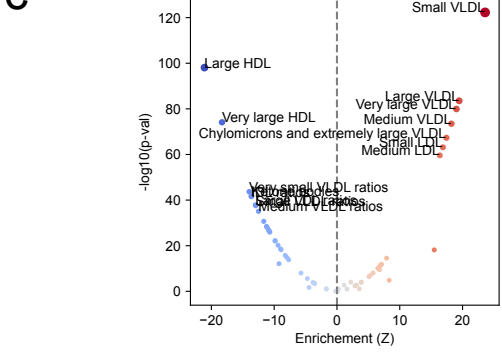

d

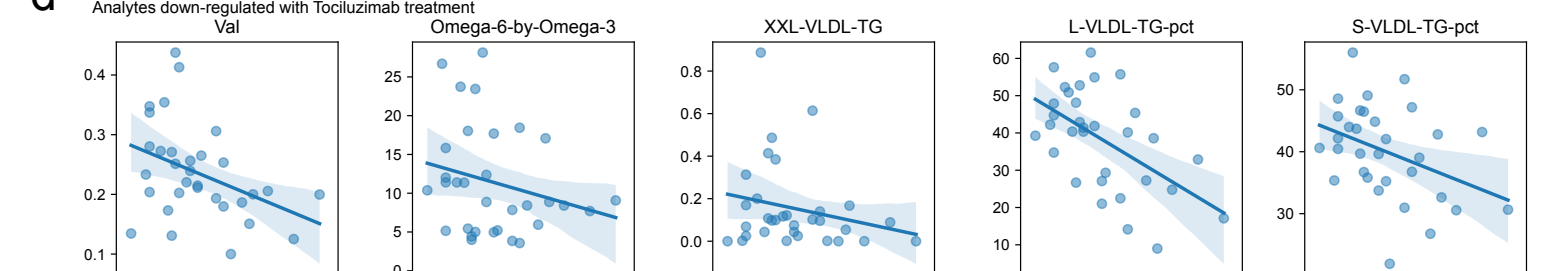

e

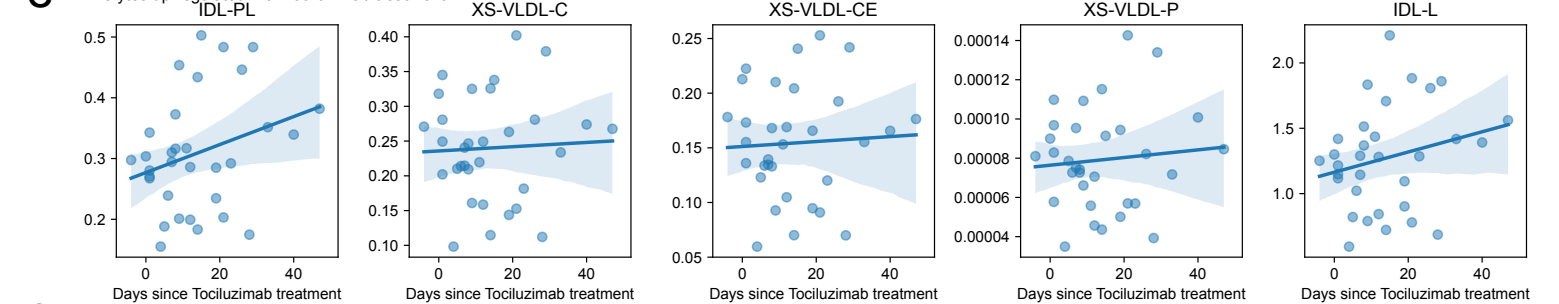

f

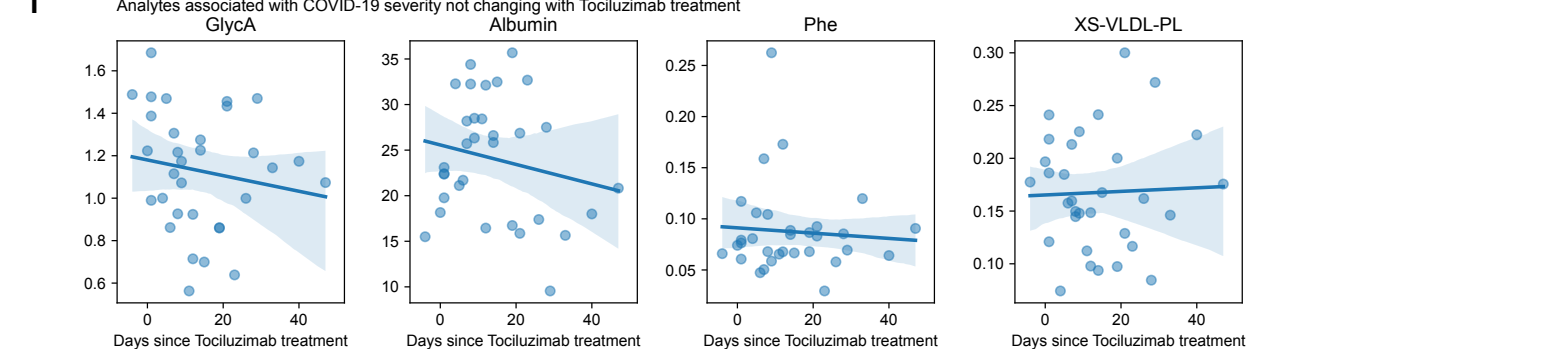

Supplement: Supplementary Figure 4 — Metabolic changes associated with tocilizumab treatment in COVID-19. (A) Association of metabolite abundance with the time of tocilizumab treatment for all metabolic species (upper panel). The lower panel illustrates the 10 metabolites most associated in each direction. (B) Heatmap of metabolites significantly associated with tocilizumab treatment for samples of patients that have been treated. Volcano plot of clinical variables associated with COVID-19 severity in our cohort. (C) Enrichment of metabolite classes in the change with tocilizumab treatment. (D–F) Abundance of metabolites with discordant (D), concordant (E) or indifferent (D) change between COVID-19 severity and tocilizumab treatment for treated patients. [file Image_4.pdf]

Supplementary Figure 5

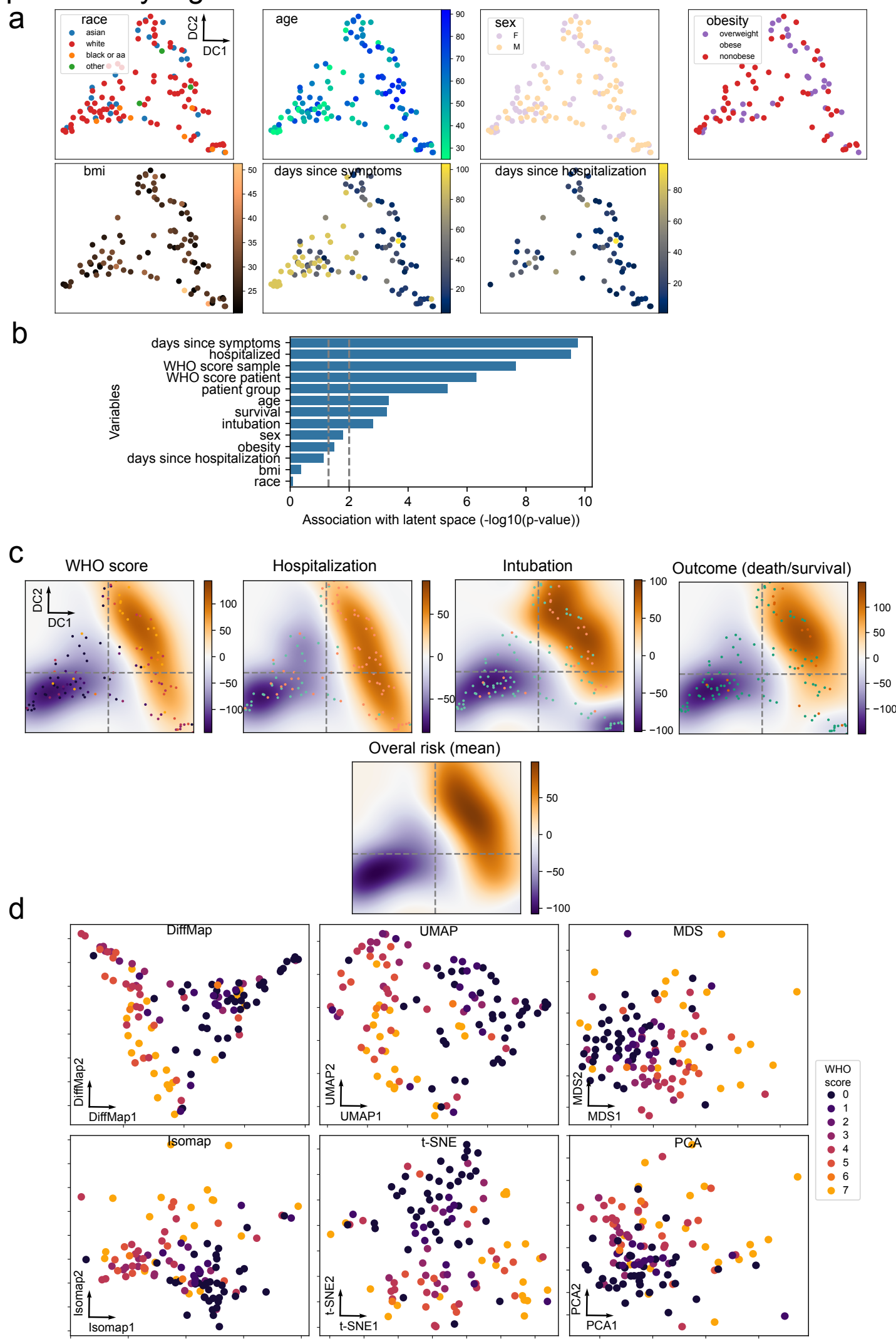

Supplement: Supplementary Figure 5 — Metabolic and clinical association of disease severity. (A) Latent space as in Figure 2A , but illustrating the distribution of additional clinical factors. (B) Association analysis of clinical variables with the latent space axes. p-values have been adjusted with the Benjamini-Hochberg FDR method. (C) Difference between bivariate kernel density estimates that have been weighted with the clinical parameters of the samples. The overall risk is the mean of the four clinical parameters in the first row of plots. (D–F) Latent space embeddings of metabolomic data using alternative methods. Samples have been colored by the WHO score scale. [file Image_5.pdf]
